# Effect of Rolling on the Microstructure and Properties of Cast Al-10Ce-4Mg Alloy

**DOI:** 10.3390/ma19142993

**Published:** 2026-07-11

**Authors:** Gaurav Singh, Humphrey Wara Odhiambo, Mohamad Hasan Bin Tasneem, Monica A. Soare, Jun Cui, Ralph E. Napolitano, Catalin R. Picu, Gaoyuan Ouyang

**Affiliations:** 1 Department of Mechanical, Aerospace, and Nuclear Engineering, Rensselaer Polytechnic Institute, Troy, NY 12180, USA; tasnem2@rpi.edu (M.H.B.T.); picuc@rpi.edu (C.R.P.); 2 Department of Materials Science & Engineering, Iowa State University, Ames, IA 50011, USA; hwara@iastate.edu (H.W.O.); cuijun@iastate.edu (J.C.); ren1@iastate.edu (R.E.N.); gaoyuan@iastate.edu (G.O.); 3 GE Aerospace Research, Niskayuna, NY 12309, USA; soare@geaerospace.com; 4 Ames National Laboratory, Division of Materials Sciences and Engineering, Ames, IA 50011, USA

**Keywords:** Al-Ce-Mg, rolling, microstructure characterization, mechanical properties

## Abstract

**Highlights:**

Al-10Ce-4Mg is studied in this work in the as-cast and rolled states, before and after exposure to 300 °C for up to 100 h. The effect of rolling on the microstructure and mechanical behavior is determined. The effect of exposure to elevated temperature on room temperature mechanical properties is shown to be weak. Testing at elevated temperatures is also performed, and it is shown that the properties of this alloy at 300 °C are superior to those of many commercial Al alloys at this temperature. These findings qualify Al-10Ce-4Mg as an excellent high-temperature Al alloy.

**What are the main findings?**

- Rolling Al-10Ce-4Mg increases tensile strength and strain at failure by ~100%. Rolling offers a simple, effective processing route to significantly enhance as-cast mechanical properties.
- Th alloy retains 83% of its strength after 100h of exposure to 300 °C.
- Strength increase is driven by higher dislocation density and texture, not grain refinement. Microstructure remains highly stable with no significant phase changes during heat treatment.
- Superior thermal stability over commercial alloys such as AA5182 at 300 °C is demonstrated. Thermodynamic stability of Al_11_Ce_3_ prevents particle coarsening at elevated temperatures.

**Abstract:**

Aluminum alloys based on the Al-Ce-Mg system, with microstructure and properties weakly sensitive to elevated temperatures, are developed for applications such as thermal engine blocks and supersonic aircraft fuselage components. Cast Al-10Ce-4Mg (wt%) was produced by slab casting and further processed by rolling. Rolling with an area reduction of 54% results in a 97% increase in strength (tensile strength of 269 MPa at room temperature) and a more than a factor of two increase in strain at failure relative to the as-cast (AC) state. We show that Al-10Ce-4Mg is highly stable upon exposure to elevated temperature: 83% strength retention after exposure to 300 °C for 100 h, without reduction in the strain at failure. The yield stress (YS) follows the same trend, increasing by ~40% upon rolling and retaining 92% of its value after exposure to 300 °C for 100 h. The ratio of the strength at 300 °C to the strength at room temperature is 0.45, which is larger than the respective ratio of most commercial Al alloys. Rolling increases the dislocation density and introduces texture, while not modifying the grain size significantly and fragmenting only the largest intermetallics. Exposure to elevated temperatures up to 100 h has no measurable effect on grain size and intermetallic distribution, while slightly changing texture. This microstructural evolution is used to rationalize the observed changes in mechanical properties.

## 1. Introduction

Al-Ce alloys have been explored for use in components like cylinder heads, turbochargers, and turbine blades calling for high strength at elevated temperatures coupled with the low density and corrosion resistance generally offered by aluminum alloys. The Al-Ce alloys are particularly noted for their stability at elevated temperatures, with a large fraction of room temperature mechanical properties being retained up to 400 °C [1,2,3,4]. This exceptional property retention can be attributed to the thermodynamic and microstructural stability of the Al_11_Ce_3_ intermetallic phase [5,6,7,8], where low Ce solubility and diffusivity in the fcc Al solution phase provide resistance to both dissolution and particle coarsening at elevated temperatures [9]. The attractiveness of Al-Ce alloys is further enhanced by the relatively low cost and availability of Ce, compared to other rare earth elements [10,11,12].

Since the mechanical strength offered by Ce addition is mainly caused by the dispersion of the Al_11_Ce_3_ intermetallic phase, these alloys generally exhibit relatively low room-temperature strength in the as-cast condition [4,13,14]. However, strength can be enhanced by alloying, e.g., using solution hardening which may remain effective at elevated temperatures. For example, the addition of Mg has been shown to significantly enhance the room-temperature strength [15], with Al-Ce-Mg alloys retaining the good casting characteristics of Al-Ce alloys.

The best performance in the binary alloy system is observed close to the eutectic composition (Al-10Ce), primarily due to the presence of fine lamellar or rod-like particles (the notation, here and subsequently, denotes wt% of the respective elements, with the balance being Al). Post solidification methods available for refinement of the intermetallic dispersion at fixed volume fraction include thermomechanical processes, such as extrusion, that may serve to fracture the lamellar and rod-like structures [14,16,17,18]. Rolling, a simpler and more broadly applicable processing technique, has been explored less in relation with Al-Ce alloys, despite its technological relevance [19,20]. Wang et al. studied the microstructure evolution upon cold rolling of Al-5Ce alloy. This is a hypoeutectic alloy with a cast microstructure of primary FCC Al plus Al_11_Ce_3_ intermetallic, which has a dendritic structure. After cold rolling (reduction rate not specified), the dendritic features were no longer discernable, and the intermetallic particles became aligned in the rolling direction [21]. Zhang et al. developed a two-phase heterogeneous lamellar structure in Al-9Ce by cold rolling, with oriented Al_11_Ce_3_ particles, fine grains, and coarse grains arranged in layers. The ultimate tensile strength (UTS) increased from 124 MPa to 200 MPa, from the as-cast to the rolled state [20]. A recent study on Al-10Ce-3Mg-5Zn alloy showed excellent properties in as-extruded and heat-treated conditions, but the ratio of UTS at 300 °C relative to the room temperature UTS was only 0.24 [22].

The present work focuses on the thermal stability and property retention at high temperature of the Al-10Ce-4Mg alloy in the cast and rolled states. To our knowledge, this is the first study of a rolled Al-Ce-Mg alloy. Microstructures are characterized using SEM, XRD and EBSD. Mechanical properties are measured at ambient and elevated temperatures by uniaxial tensile testing. We examine the structural refinement effects of rolling on mechanical properties, as well as thermal stability, by room temperature mechanical testing in as-cast and hot-rolled conditions, before and after exposure to elevated temperatures. We observe that rolling produces texture and an increase in the dislocation density, which lead to increased yield stress, UTS and ductility relative to the cast state. Exposure to elevated temperatures has weak effects on the microstructure and mechanical behavior.

## 2. Materials and Methods

**Figure 1:**
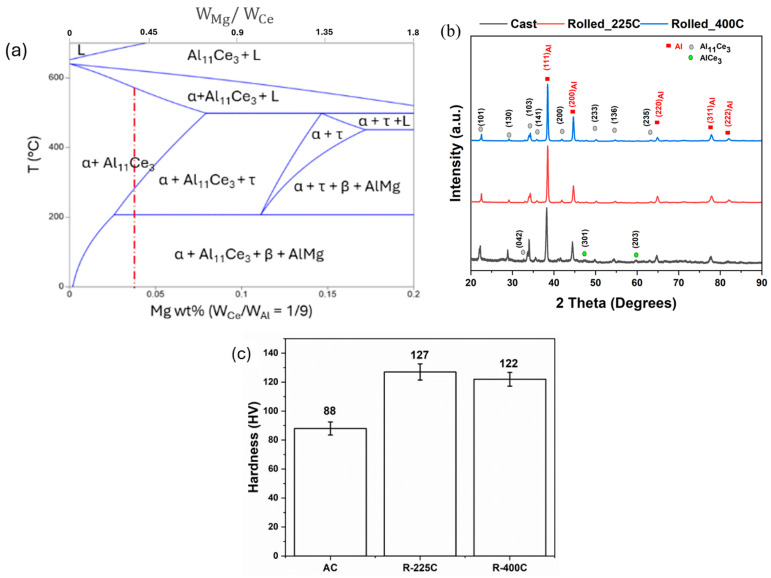
(**a**) Calculated pseudo-binary Al–Ce–Mg phase diagram. The 4%Mg composition considered in this study is indicated by the red line. (**b**) XRD patterns for AC and rolled states Al-10Ce-4Mg alloy. (**c**) Room temperature Vickers hardness of AC and rolled samples.

Al-10Ce-4Mg alloys were prepared by combining Al-20Ce and Al-50Mg master alloys with pure (0.9999 by weight) Al at 850 °C in a graphite crucible, followed by mixing and casting into a graphite mold preheated to 400 °C. The elevated melting temperature was used to ensure complete dissolution of the Al-20Ce master alloy and to achieve homogeneous mixing of Ce and Mg in the melt, consistent with established practice for experimental Al-Ce-Mg alloys [18,23]. The liquidus temperature of Al-10Ce-4Mg is approximately 640–660 °C based on the Thermo-Calc phase diagram (Figure 1a); optimized commercial processing would use temperatures closer to this range. Cast Al-10Ce-4Mg ingots (width of 65 mm, thickness of 16 mm, and length of 430mm) were rolled either at 225 °C or at 400 °C. These temperatures were selected to span the τ phase (Al_13_CeMg_6_) solvus, as shown in the calculated pseudo-binary Al-Ce-Mg phase diagram of Figure 1a (obtained with Thermo-Calc [24,25]). The composition used here (4% Mg) is indicated by the red line.

Cast samples were heated to the target rolling temperature and equilibrated for 30 min before they were removed from the furnace and immediately fed to the rolling mill. The rolls were not heated. The thickness reduction was 10% for each pass, and samples were reheated to the rolling temperature prior to each subsequent pass. The total rolling thickness reduction was 54% (6 passes with 10% reduction each) in all cases. The cast ingot had an initial thickness of 16.0 mm and was hot rolled to a final thickness of 7.14 mm, corresponding to a total thickness reduction of 54%. Applying larger reductions led to excessive cracking. This reduction corresponds to a plastic strain of 61%. The rolled states are denoted by R-225C and R-400C, while AC denotes the as-cast state. The final thickness of the rolled plates was between 25 and 30mm.

Heat treatment of 10 h and 100 h at 300 °C was applied to selected samples, which were subsequently tested at room temperature to evaluate property retention.

Tensile specimens with a 52 mm gauge length and cross-sectional dimensions of 5 mm × 6 mm were machined per the ASTM E8 standard [26] for all cast, rolled, and heat-treated conditions. Tensile tests were performed using an Instron machine (Instron 5969, 50 kN load cell) under quasi-static conditions ($\dot{\varepsilon \;}$= 3 × $10^{-3}$ s^−1^) at room temperature (25 °C). Tests at elevated temperatures of 150 °C, 200 °C, 250 °C, 300 °C and 350 °C were performed with the same system on samples that were cast and rolled but not heat treated. For the at-temperature tests, samples were heated at a rate of 5 °C/s to reach the test temperature, followed by a 300 s hold before the beginning of the test.

Hardness was measured at room temperature with a Vickers microhardness tester (Qness 60M QATM) by applying a load of 500 g, with 15 s dwell time, in accordance with ASTM E384-17 [27]. Ten measurements were made for each sample condition.

A FEI SEM was used for metallography and fractography studies. Microstructural imaging was performed using a Backscattered Electron (BSE) detector, which provides atomic number contrast to distinguish the Ce-rich Al_11_Ce_3_ intermetallics (bright) from the Al matrix (dark). Polished planar surfaces were prepared for examination by etching with Kellar’s reagent for 10 s. EBSD was performed using an Oxford SEM microscope (Oxford Instruments plc, High Wycombe, UK). Specimens were subjected to extra polishing steps (diamond and colloidal silica) to increase surface planarity. EBSD image analysis was carried out using the MTEX 5.11.2 software. Phase mapping is based on FCC Al and orthorhombic Al_11_Ce_3_.

X-ray diffraction (XRD) measurements were performed on a Bruker DB-Discover (manufactured by Bruker AXS GmbH, Karlsruhe, Germany) using Cu Kα radiation (λ = 0.15406 nm), operating at 45 kV and 40 mA. Scans were conducted over a 2θ range of 20° to 90°, with a step size of 0.02° and a scanning rate of 2° min^−1^. Peak indexing was performed with reference to ICDD PDF #04-002-3407 for FCC Al and ICDD PDF #47-1070 for Al_11_Ce_3_ (orthorhombic, space group Immm, No. 71).

## 3. Results and Discussion

Figure 1b shows the normalized XRD diagram demonstrating the presence of FCC Al, AlCe_3_ and Al_11_Ce_3_ phases in both AC and rolled conditions. No additional phase is discernable from XRD in these samples. A slight evolution of the Al peaks is observed between the AC and the two rolled states indicating texture evolution, with the difference becoming more pronounced with increasing rolling temperature. The data shows no indication of phase composition changes associated with rolling, nor evidence of the ternary τ phase (or any other Mg-bearing phase) predicted by Thermo-Calc, as shown in the phase diagram of Figure 1a. The absence of the τ phase is attributed to the fact that the volume fraction of τ at this composition may fall below the practical XRD detection limit.

Hardness measurements were performed on AC and rolled samples, and the results are shown in Figure 1c. Hardness increases upon rolling, as expected, but is identical in the two rolled states, R-225C and R-400C.

Based on these observations—particularly considering the lack of evidence for the existence of the τ-phase—we selected R-400C Al-10Ce-4Mg for further mechanical testing and characterization. Samples were tested in the as rolled (AR) state and were evaluated for property retention at room temperature after exposure to 300 °C for 10 h (AR-10) and 100 h (AR-100).

**Figure 2:**
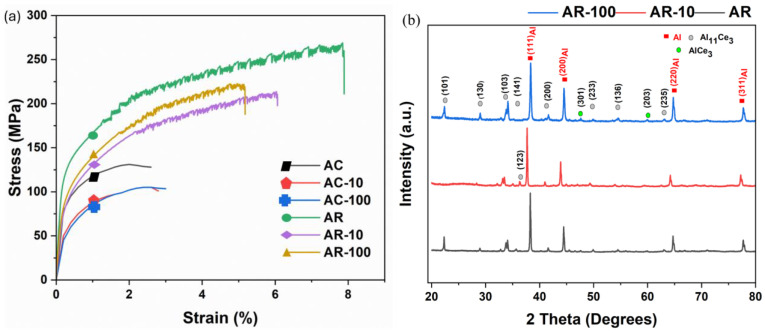
(**a**) Engineering stress–strain curves for as-cast and rolled samples before and after heat treatment. (**b**) XRD spectra of rolled samples before and after heat treatment. 3 samples are tested for each condition, and these are representative curves.

The engineering stress–strain curves of as-cast and cast and rolled samples, before and after heat treatment, are shown in Figure 2a. Comparing AC and AR, it may be seen that rolling greatly improves mechanical properties: the yield stress increases by ~40%, while UTS and the strain at failure both increase by about 100% (Table 1). The stress–strain curves of the rolled samples (Figure 2a) exhibit serrated flow (Portevin-LeChatelier, PLC, effect), indicating the occurrence of dynamic strain aging (DSA), which is known to take place in Al–Mg alloys [28,29]. DSA arises from the interaction between diffusing Mg solute atoms and dislocations [30,31], leading to intermittent pinning and unpinning events that trigger plastic deformation instabilities which, in turn, manifest macroscopically as serrations of the stress–strain curves. This process increases the effective flow stress and contributes to strengthening. Plastic deformation during rolling also introduces a high density of geometrically necessary and statistically stored dislocations, further elevating the yield stress and UTS. Serrations do not show up in the cast case because these samples break before the incubation strain (strain at the onset of the PLC effect). This is consistent with the incubation strain of ~3–5% reported for AA5182-O (Al-4.5Mg) under quasi-static loading at room temperature [29], which exceeds the failure strain of the as-cast Al-10Ce-4Mg (~2.6%, Table 1).

Exposure to 300 °C for 10 h and 100 h leads to the reduction of the yield stress and UTS in both cast and rolled states, as shown in Table 1. In the AC case, UTS after 10 h is the same as that after 100 h of annealing and ~20% lower than the UTS of the AR state. The AR-10 and AR-100 curves (Figure 2a) show a decrease in the UTS relative to the AR state of 20% and 17%, respectively. The yield and UTS retention (ratio of AR property to that after heat treatment) were measured to be 77% and 80% for AR-10, and 92% and 83% for AR-100, respectively. In other words, the YS is largely retained upon exposure to elevated temperature, with the AR-100 state recovering to 92% of the as-rolled YS, exceeding the corresponding UTS retention (83%). The same trend holds in the as-cast state, where the YS retention is 70% (AC-10) and 81% (AC-100), comparable to the UTS retention of 77% and 82%, respectively (Table 1). A concomitant decrease in elongation at failure is observed, with values decreasing to 77% and 66% of the AR values for AR-10 and AR-100, respectively.

Further, we investigate the microstructural origins of the observed effects of rolling and heat treatment. The XRD pattern of heat-treated and non-heat-treated samples is shown in Figure 2b. No phase change was observed to occur during heat treatment across different exposure times. Changes in the Al (200) peaks are observed as the samples go from AR to AR-100, indicating texture variation, as discussed further via EBSD analysis.

**Figure 3:**
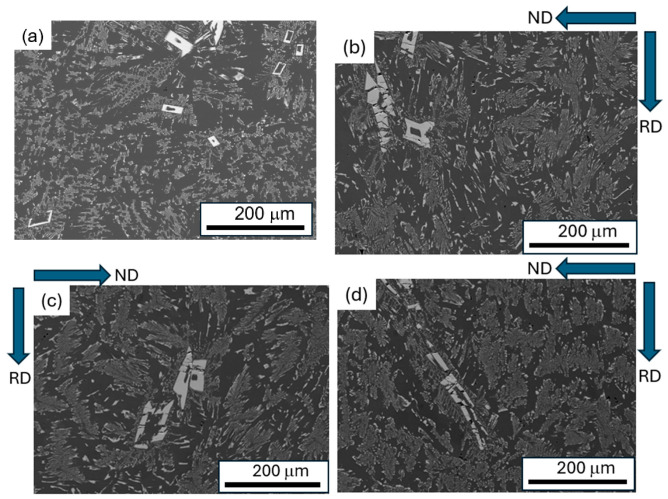
The microstructure of (**a**) AC, (**b**) AR, (**c**) AR-10, and (**d**) AR-100 (black = Al, white = Al_11_Ce_3_, lighter shade = eutectic).

Figure 3a–d shows SEM images of the microstructure of AC, AR, AR-10 and AR-100 sample states. The AC microstructure shown in Figure 3a exhibits dendritic and blocky intermetallics. The dendrites are reminiscent of the eutectic structure. The binary eutectic has 10% Ce. Mg shifts the eutectic point to slightly higher Ce concentrations [32] and hence at 10% Ce the material does not exhibit the characteristic eutectic microstructure of the binary Al-10Ce alloy. Rolling does not lead to significant changes in the overall intermetallic structure. The larger, blocky intermetallics are fragmented, but the dendritic structure is not affected by the rolling reduction applied. Further, comparing the AR structure of Figure 3b with the rolled and heat-treated structures of Figure 3c,d, it is concluded that annealing has no visible effect on the microstructure observed by SEM.

**Figure 4:**
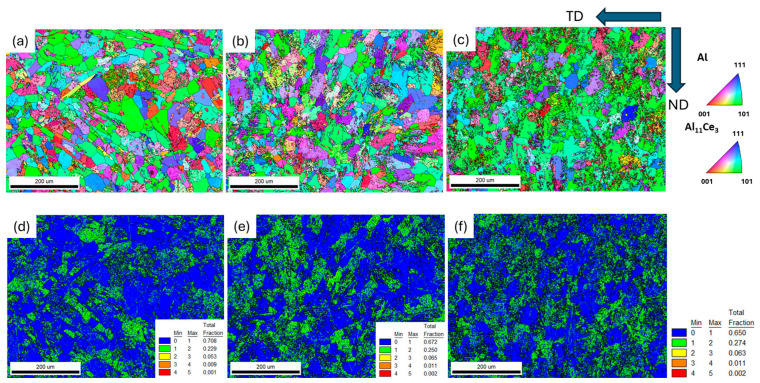
Inverse pole figure (IPF) map of (**a**) AR, (**b**) AR-10, (**c**) AR-100, and KAM of (**d**) AR, (**e**) AR-10, and (**f**) AR-100 for Al-10Ce-4Mg samples. (TD is transverse direction, ND is normal direction). In KAM Map, the blue corresponds to low local misorientation (0–1°), while green, yellow, orange, and red represent progressively higher local misorientation (1–2°, 2–3°, 3–4°, and 4–5°, respectively), which are indicative of increasing local lattice distortion and geometrically necessary dislocation density.

Figure 4 shows results from the EBSD analysis. Figure 4a–c show the EBSD inverse pole figures for AR, AR-10, and AR-100 samples. The grain size distribution may be inferred from these measurements. The mean grain size of AR, AR-10 and AR-100 is 27 ± 3 μm, 26 ± 2 μm and 23 ± 2 μm, respectively. For reference, the grain size of AC samples is 24 ± 1 μm. Hence, rolling with 54% reduction, as used here, does not lead to grain refinement. A similar conclusion was obtained when rolling AA5754 [33] and AA5182 [34] with reductions of 56% and 50%, respectively.

**Figure 5:**
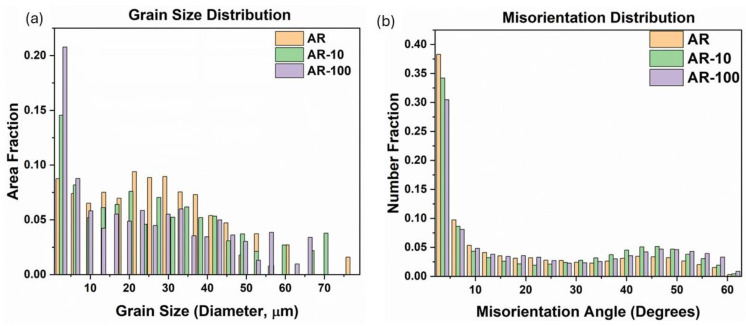
(**a**) Grain size distribution and (**b**) misorientation distribution of AR, AR-10, and AR-100 for Al-10Ce-4Mg samples.

While the mean grain size is approximately identical in the various material states considered, the distribution of grain sizes shown in Figure 5a provides additional information. Comparing AR with AR-100, one observes an increase in the fraction of small grains and a corresponding decrease in the fraction of medium and large grains. This indicates a weak recrystallization trend. The effect is not pronounced enough (upon 100 h of exposure to 300 °C) to significantly modify the mean of the distribution. Interestingly, no evidence of intermetallic evolution is provided by SEM, Figure 3, and hence the emergence of small recrystallized grains indicates that Zener pinning is insufficient to prevent grain boundary migration.

Figure 4 also provides texture information. As expected, rolling introduces texture (compare AC with AR). The texture is weakly sensitive to heat treatment and has a non-monotonic variation with the annealing time. The maximum texture intensity is reported in Table 2 for AR, AR-10 and AR-100 samples. The texture decreases upon annealing for 10 h and then increases after 100 h of annealing. The same trend is observed in the variation of the flow stress and UTS with the annealing time when comparing AR, AR-10 and AR-100 samples, Figure 2a.

The kernel average misorientation (KAM) maps corresponding to the structures in Figure 4a–c are shown in Figure 4d–f. This provides the distribution of misorientations which is shown in Figure 5b. The distribution shows a weak decrease in the fraction of small angle boundaries and a corresponding increase in the fraction of large angle boundaries. This supports the conclusion that some degree of recrystallization takes place, as suggested by the grain size analysis, Figure 5a. However, this trend is weak, and the distribution shape remains unchanged, pointing to the stability of the microstructure upon exposure to elevated temperatures. The fraction of high angle (HAGB) and low angle grain boundaries (LAGB) is shown in Table 2 for the three states of Figure 4. The threshold used to define the two categories of boundaries is 15 degrees. Table 2 also shows the average misorientation (average of the distribution in Figure 5b).

The average KAM value is used to compute the dislocation density (geometrically necessary dislocations) based on the strain gradient approach and the expression:

(1) $$\rho_{GND}=\frac{2\theta }{n\lambda \left|b_{D}\right|}$$

where θ is the KAM value, λ is the step size (1 µm), n is the number of nearest neighbors in the KAM calculations which is five in this case, and $b_{D}$ is the Burgers vector for Al, $b_{D}$ = 0.286 nm. The dislocation density values predicted by Equation (1) are shown in Table 2.

These microstructural observations may be used to rationalize the mechanical behavior shown in Figure 2a. This discussion has two parts: (i) the effect of rolling, and (ii) the effect of heat treatment. As discussed above, rolling has a marginal effect on grain size and dispersoid size and distribution but introduces texture and increases the dislocation density by a factor of ~2, as shown in Table 2. The increase in dislocation density correlates with the 43% increase in yield stress from the AC to AR states, as shown in Table 1. Specifically, the yield stress is expected to increase with the square root of the dislocation density, i.e., by a factor of $\sqrt{2}$ between these two states. The hardening rates of AC and AR are similar, which is expected since the intermetallic microstructure remains unchanged. The UTS increases significantly in the AR case since failure is delayed. The increase in ductility upon rolling may be attributable, in part, to the closure of casting-induced pores, which can act as stress concentrators and sites of premature failure initiation in the as-cast material. Although we observe occasional pores in the AC material, porosity quantification was not performed in this study and hence the proposed argument for the lower performance of the AC samples remains a conjecture. The ductility increase is also promoted by the developing texture and increased dislocation density introduced by rolling.

The heat treatment produces a limited degree of recrystallization manifested as an increase in the fraction of small grains and of the fraction of high angle grain boundaries. The dispersoid distribution and size are not affected by the applied heat treatment to a measurable degree. Hence, we associate the reduction in the flow stress and UTS upon heat treatment with recrystallization and the weak evolution of texture.

**Figure 6:**
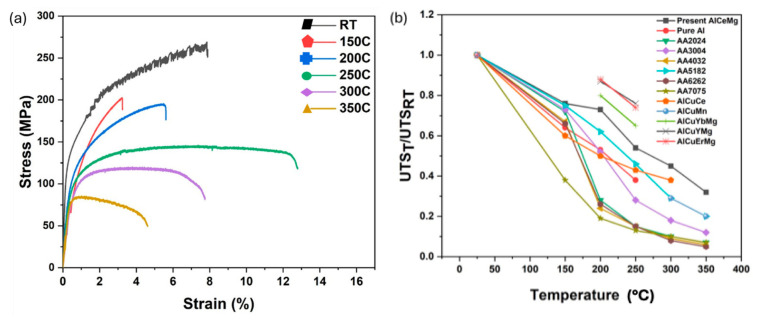
(**a**) Stress–strain curves for Al-10Ce-4Mg in the AR state tested at various temperatures. (**b**) Variation with temperature of UTS compared with several commercial Al alloy [37,38,39,40,41,42].

Further, we investigate the mechanical behavior at elevated temperatures. Tests were performed at 150 °C, 200 °C, 250 °C, 300 °C and 350 °C. Figure 6a shows stress–strain curves obtained at various temperatures. The strength and yield stress decrease as the temperature increases, as expected. The PLC effect is not observed above room temperature. In fact, PLC is generally observed only in a range of temperatures and strain rates [35] and the data in Figure 6a indicates that, in the case of the material studied here, the boundary of the temperature range in which PLC is active is between room temperature and 150 °C. The DSA driving the PLC effect in this alloy arises from the interaction between mobile Mg solute atoms remaining in solid solution and gliding dislocations. Multiple DSA mechanisms have been proposed, all being based on the transient clustering of mobile solute atoms to arrested mobile [30,35,36] or forest [31] dislocations. These clusters cause pinning and increase the flow stress required to restart dislocation motion. These dynamics and associated critical stress fluctuation were used to explain the PLC effect. The increased dislocation density introduced by rolling increases the likelihood of DSA and PLC relative to the as-cast state. The size and dynamics of solute cluster formation are affected as temperature increases, reducing the probability of DSA, consistent with the disappearance of serrations above room temperature in Figure 6a.

Figure 6b shows the variation with temperature of the UTS of AR Al-10Ce-4Mg together with data for several commercial Al alloys (pure Al, AA2024, AA3004, AA4032, AA5182, AA6262, and AA7075) [37]. Al-10Ce-4Mg has a ratio of the UTS at 300 °C to the UTS at room temperature of 0.45. It is the largest value of all alloys shown; the respective ratio for the commercial alloys is 0.1 for AA2024, 0.18 for AA3004, 0.1 for AA4032, 0.29 for AA5182, 0.08 for AA6262, to 0.1 for AA7075. Recently developed Al-Cu-REM alloys (e.g., Al-Cu-Y-Mg and Al-Cu-Er-Mg [38]) exhibit higher normalized strength retention at intermediate temperatures (200–250 °C). However, these alloys generally possess very limited room-temperature ductility (typically below 1% elongation), whereas the present Al-10Ce-4Mg alloy exhibits substantially higher ductility (2.6% in the as-cast condition and 7.9% after rolling). Thus, the present alloy offers a more favorable balance between elevated-temperature strength retention and tensile ductility, which is advantageous for structural applications requiring both strength and formability. The commercial alloy AA5182-O (Tempered) is included as reference since its composition is approximately Al-4Mg, which is close to the alloy considered here (if one excludes Ce). The reduction in the UTS in the case of AA5182 is due to the dissolution of fine AlMg precipitates [39]. The coefficient of variation of the UTS for Al-10Ce-4Mg across all conditions is between 2% and 5%. Al-10Ce-4Mg exhibits improved behavior as compared to AA5182 due to the stability of the Al_11_Ce_3_ dispersion. The corresponding curves for the commercial high temperature alloys AA2024 and AA7075 are also included [37]. While the room temperature UTS of these alloys is larger than that of the other alloys included in this comparison, their UTS at 300 °C is approximately half of the UTS of Al-10Ce-4Mg (AR) at the same temperature.

## 4. Conclusions

The effect of rolling on the mechanical behavior of the ternary Al-10Ce-4Mg was studied and compared with the behavior in the as-cast state. Rolling at 400 °C with 54% reduction (the largest before cracking develops during processing) causes a drastic change in the mechanical behavior relative to the cast state: both UTS and the strain at failure increase by ~100%. The yield stress, the more structure-sensitive and commercially relevant strength measure, increases by ~40% upon rolling. Upon exposure to 300 °C for durations up to 100 h, UTS decreases by ~20% in both rolled and cast states. The yield stress is similarly stable, with the rolled alloy retaining 92% of its as-rolled YS after 100 h at 300 °C. These observations are related to the state of the microstructure in the cast, rolled and heat-treated states. Both rolling and heat treatment leave the mean grain size and Al_11_Ce_3_ dispersoid structure unchanged. Rolling increases the dislocation density, introduces texture and possibly closes pores and defects that may cause premature failure in the cast material, therefore increasing the yield stress, the UTS and the ductility. Heat treatment leads to weak recrystallization and slight variation in texture, which causes the observed flow stress reduction. Dynamic strain aging is observed at room temperature in the rolled state due to the presence of Mg in solid solution. Tests at elevated temperatures demonstrate that Al-10Ce-4Mg has superior thermal stability to many commercial Al alloys.

## Figures and Tables

**Table 1:** Comparison of room temperature mechanical properties of as-cast and rolled Al-10Ce-4Mg alloy. The percentage represents strength and elongation retention after exposure to 300 °C.

	AC	AC-10	AC-100	AR	AR-10	AR-100
YS (MPa)	94 ± 2	65 ± 1 (70%)	76 ± 1 (81%)	135 ± 2	104 ± 1 (77%)	124 ± 2 (92%)
UTS (MPa)	132 ± 5	102 ± 3 (77%)	108 ± 3 (82%)	269 ± 4	214 ± 4 (80%)	223 ± 4 (83%)
% EL	2.6 ± 0.1	2.8 ± 0.1	3.0 ± 0.1	7.9 ± 0.5	6.1 ± 0.3	5.2 ± 0.2

**Table 2:** Comparison of grain size, LAGB, HAGB, average misorientation (MO), maximum texture intensity, and dislocation density estimate of as-cast (AC) and rolled (AR, AR-10, AR-100) alloy Al-10Ce-4Mg.

	Grain Size (µm)	LAGB	HAGB	Average MO	Maximum Texture Intensity	Dislocation Density (m^−2^)
As Cast	24 ± 1	62.7	37.3	20.34	-	6.92 × 10^13^
AR	27 ± 3	58.5	41.5	18.07	7.70	1.2 × 10^14^
AR-10	26 ± 2	51.1	48.8	21.90	4.90	1.15 × 10^14^
AR-100	26 ± 2	48.0	52.0	22.88	7.67	1.2 × 10^14^

## Data Availability

The original contributions presented in this study are included in the article. Further inquiries can be directed to the corresponding author.
